# Unmasking the Unseen: Dupilumab‐Induced Sarcoidosis in a Young Patient—A Case Report

**DOI:** 10.1155/crpu/3325053

**Published:** 2026-04-24

**Authors:** Mariana Issawy, Nael Bsoul, Omar Hakrush, Ahmad Igbaria, Yosef Imtanis, Amir Bieber, Lee Hilary Goldstein

**Affiliations:** ^1^ Internal Medicine Department C, HaEmek Medical Center, Afula, Israel, haemek.co.il; ^2^ Pulmonology Unit, HaEmek Medical Center, Afula, Israel, haemek.co.il; ^3^ Rheumatology Unit, HaEmek Medical Center, Afula, Israel, haemek.co.il; ^4^ The Bruce Rappaport School of Medicine, Technion, Haifa, Israel, technion.ac.il

**Keywords:** case report, drug-induced sarcoidosis-like reaction (DISR), dupilumab, granulomatous inflammation

## Abstract

Sarcoidosis is a multisystemic disease characterized by the formation of noncaseating granulomas in various organs. Drug‐induced sarcoidosis‐like reactions (DISR) mimic sarcoidosis both clinically and histologically, with numerous drugs identified as potential triggers. This case report presents a 35‐year‐old female with atopic asthma who developed DISR following treatment with Dupilumab, a monoclonal antibody targeting interleukin‐4 and interleukin‐13. The patient experienced diffuse arthralgia, myalgia, erythema nodosum, and recurrent abscesses shortly after initiating Dupilumab. Diagnostic workup, including biopsy, revealed noncaseating granulomas, confirming the diagnosis of sarcoidosis. Symptoms improved after dupilumab discontinuation, highlighting the importance of recognizing DISR in patients undergoing immune‐modulating therapy. This case report underscores the need for awareness among clinicians of the potential for sarcoidosis‐like reactions in patients treated with newer biologic agents and calls for further studies to elucidate the underlying mechanisms and management strategies for such reactions.

## 1. Introduction

Sarcoidosis is a multisystemic disease of unknown etiology, characterized by the formation of noncaseating granulomas affecting nearly any organ [1, 2]. It can be more common in African American women and among Northern Europeans. Although it frequently occurs in individuals aged 30–50, it can also affect older adults, suggesting a broader age distribution than previously thought [3, 4].

Various drugs can induce a systemic granulomatous reaction, clinically and histologically indistinguishable from sarcoidosis. These reactions are termed drug‐induced sarcoidosis‐like reactions (DISR) [5, 6]. According to recent reports, drugs commonly associated with DISRs include immune checkpoint inhibitors, highly active antiretroviral therapy, interferons, tumor necrosis factor‐*α* antagonists, and other miscellaneous drug classes [6]. However, new reports frequently emerge about novel drugs that may also cause DISRs. The pathogenesis of DISR is not well understood. It is unclear whether these drugs directly cause sarcoidosis, facilitate its development by affecting the immune system, or possibly reactivate latent sarcoidosis [6, 7].

Similar to sarcoidosis, most cases of DISRs do not require treatment, as they generally cause minimal symptoms with no organ damage [7, 8]. However, when treatment is necessary, drugs used for sarcoidosis (immunosuppressants) can be effective. Moreover, studies have shown that withdrawing the causative agent tends to resolve DISR cases [6].

This case report describes DIST associated with dupilumab, which should be added to the list of those associated with DISR. It also describes a case of DISR with atypical symptoms underscoring the importance of considering DISR in the differential diagnosis of rheumatological conditions.

## 2. Case Report

A 35‐year‐old female with a history of atopic asthma, treated with corticosteroids (prednisone 20 mg) for the previous 9 years presented with diffuse arthralgia, myalgia, and a generalized rash on both legs, along with recurrent axillary abscesses; all of which appeared after starting treatment with dupilumab (a monoclonal antibody blocking interleukin‐4 [IL‐4] and interleukin‐13 [IL‐13]) 3 months prior. During this time, prednisone dose was tapered down to 2.5 mg per day.

Her physical examination was remarkable for an erythema nodosum‐like rash on both legs. Laboratory results showed a leukocyte count within normal limits, hemoglobin 10.4 mg/dL, and normal serum creatinine, liver function tests and electrolytes, including calcium and 25‐hydroxy vitamin D levels. C‐reactive protein was elevated at 11.7 mg/dL. Angiotensin‐converting enzyme was 30.5 U/L, within normal limits. Serology tests including ANA, antidouble stranded DNA, anti‐Smith, anticentromere, anti‐Scl‐70, anti‐CCP, anti Jo‐1, antiproteinase 3, anti‐myeloperoxidase as well as HIV all were negative.

**Figure 2:**
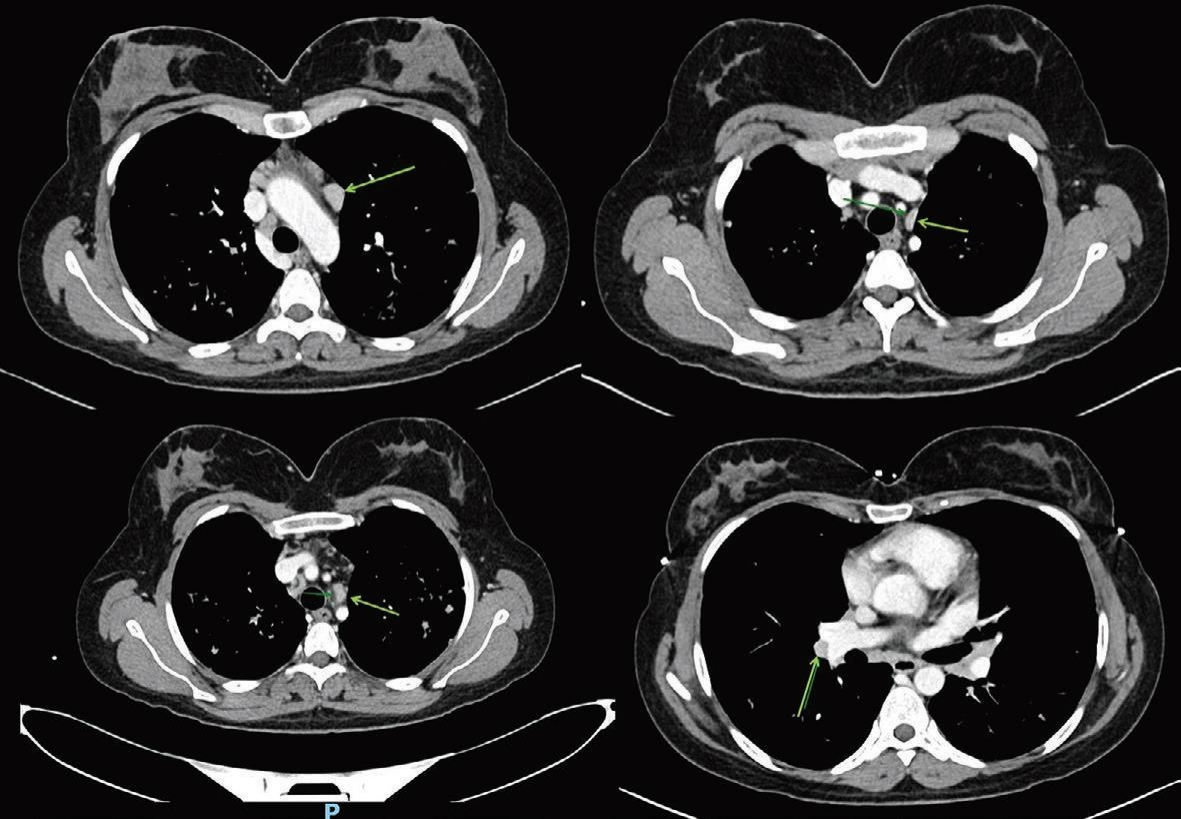
Axial mediastinal‐window chest CT images, demonstrating mild bilateral hilar and mediastinal lymphadenopathy.

**Figure 3:**
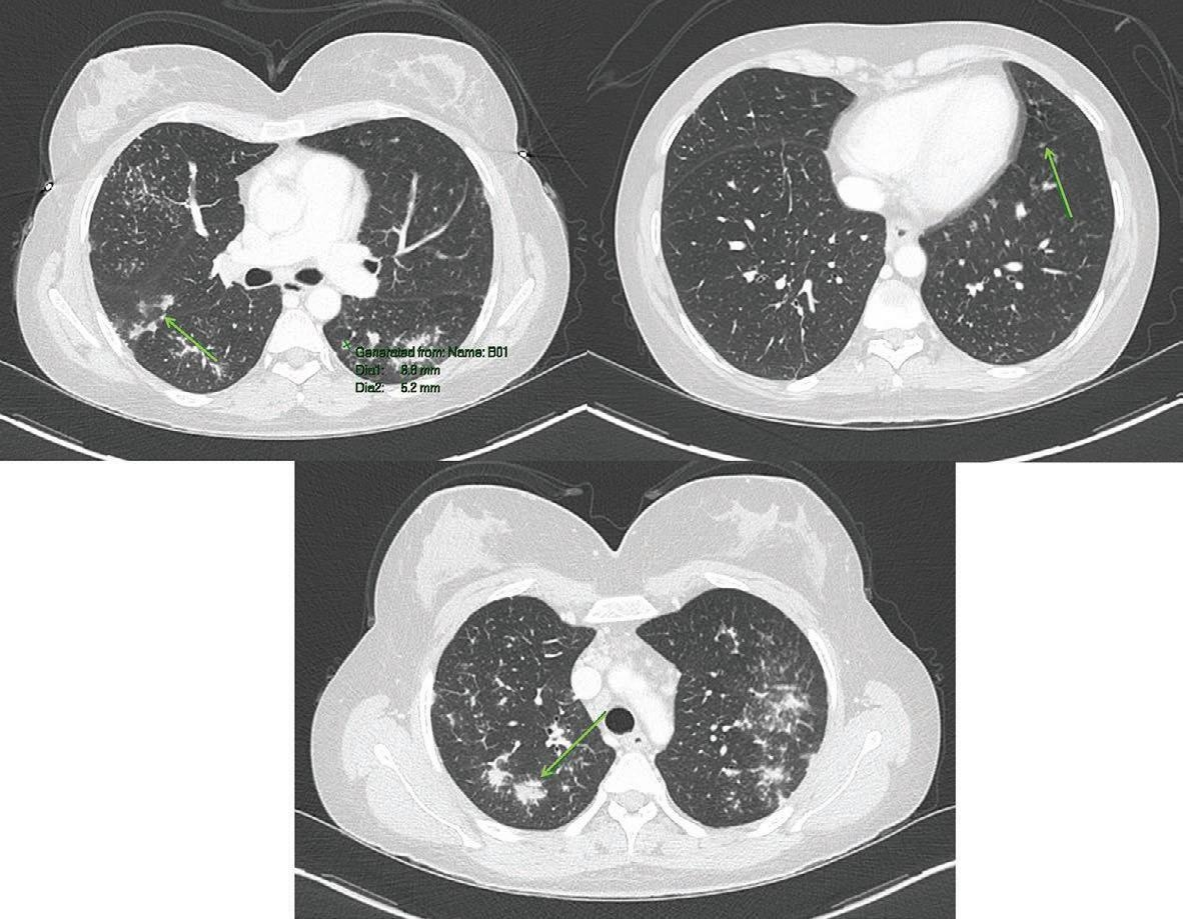
Axial lung‐window chest CT images, demonstrating multiple peribronchovascular and perilymphatic nodular opacities.

A skin biopsy was taken. Chest x‐rays did not show hilar lymphadenopathy but suggested subtle reticulonodular changes (Figure 1). A total body computed tomography (CT) scan revealed suspicious findings of sarcoidosis including multiple small alveolar opacities and diffuse nodular opacities, primarily in a peribronchovascular and perilymphatic distribution. Enlarged lymph nodes in both lung hila, the largest measuring 1.2 cm in diameter. In the mediastinum, several lymph nodes of borderline size, the largest measuring 1.1 cm in diameter (Figures 2 and 3). Bronchoalveolar lavage ruled out infectious etiology with negative PCR results and negative cultures. Transbronchial biopsy from the right upper lobe showed nonnecrotizing granulomatous inflammation. Skin biopsy revealed ruptured folliculitis in the late stage, with evidence of calcification, indicating a chronic inflammatory process. It is worth noting that echocardiography and ophthalmological evaluations did not demonstrate cardiac or ocular involvement, respectively.

Figure 1(a) December 2023—Baseline film showing clear lung fields and normal hilar contours. (b) June 2024—New bilateral reticulonodular opacities and mild hilar prominence.

**Figure:**
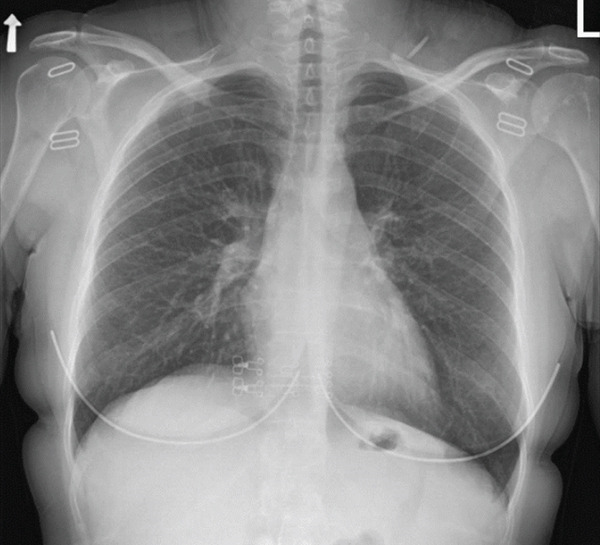
(a)

**Figure:**
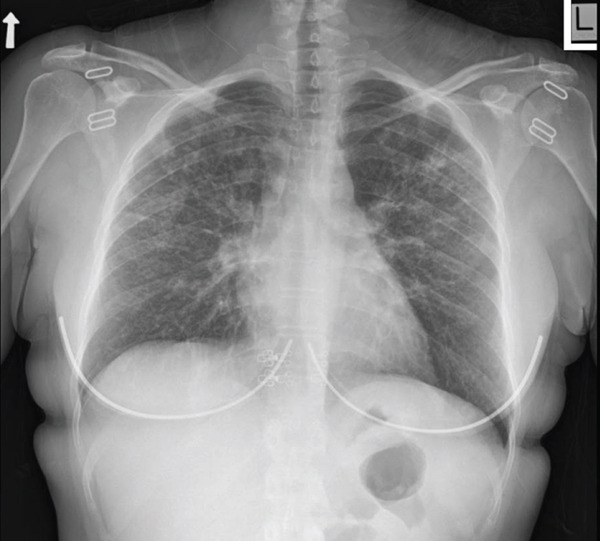
(b)

**Figure 4:**
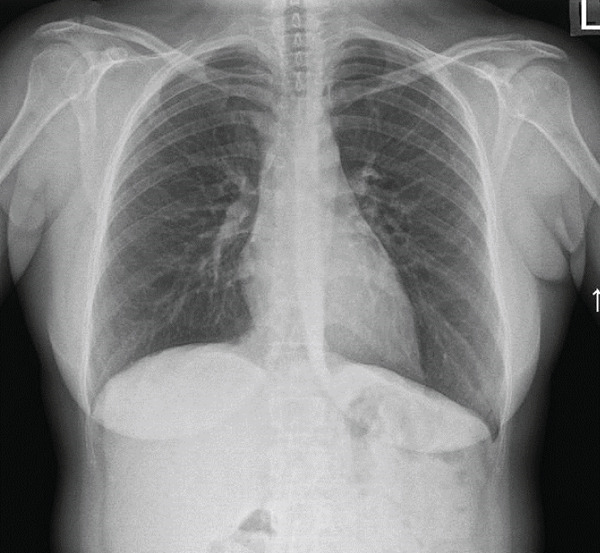
Follow‐up chest radiograph after treatment (September 2024).

Dupixent was discontinued during the diagnostic process and was not restarted. After ruling out infectious etiology, the patient restarted prednisone therapy with a starting dose of 40 mg per day, and further rather rapid reduction to 15 mg per day after weeks, due to concerns of side effects. A notable improvement in her symptoms was reported at follow‐up a couple of months afterwards (Figure 4). As for her status at the moment of this report, the option of steroid‐sparing therapy, mostly for controlling her atopy, is being considered.

## 3. Discussion

This case report presents a unique case of DISR in a young patient following treatment with dupilumab, a monoclonal antibody that blocks IL‐4 and IL‐13 [9]. Although DISRs have been previously reported with various medications, the association with dupilumab adds a novel dimension to our understanding of this condition. This is consistent with a case described by Balhoumme el at. (2020), where a 28‐year‐old male developed sarcoid‐like granulomatosis following dupilumab therapy, which was resolved after discontinuation of the drug and corticosteroid therapy [10]. Furthermore, Saito et al. (2023) documented a similar case where the patient improved without the need for immunosuppressants after dupilumab cessation [11].

The pathogenesis of DISR is not fully understood but it is thought to involve immune modulation that either triggers sarcoidosis‐like granulomas or reactivates latent sarcoidosis [6, 7]. In this case, the patient developed symptoms typical of sarcoidosis, including erythema nodosum, following the initiation of Dupixent. This temporal relationship suggests a possible causative role of Dupixent in the development of granulomatous inflammation. As Dupixent modulates the immune response by blocking key cytokines involved in the inflammatory pathway, particularly IL‐4 and IL‐13, which may play central roles in Th2‐mediated immune responses, it could potentially disrupt the balance of immune regulation, leading to granuloma formation [12]. The role of IL‐4 and IL‐13 is known to suppress Th1‐driven immunity and play a role in neutrophil recruitment and function, which is crucial for granuloma maintenance and formation in sarcoidosis [13]. By inhibiting IL‐4 and IL‐13, dupilumab may inadvertently promote a shift towards a Th1‐dominant response, resulting in the enhanced production of cytokines such as IFN‐*γ* and TNF‐*α*, which are pivotal in granuloma formation and maintenance [13, 14]. This pathophysiology of granuloma formation and neutrophil suppression was shown in previous studies, mainly in parasitic infectious hepatic reactions. It is possible that dupilumab enhanced granuloma formation, whereas another undiagnosed trigger contributed to its development [12, 14, 15].

The clinical presentation of DISR in this patient, marked by diffuse arthralgia, myalgia, rash, and recurrent abscesses, underscores the variability in manifestations of drug‐induced granulomatous reactions. The presence of erythema nodosum, a common dermatological manifestation of sarcoidosis, was a key clinical point in this patient′s presentation and raised the suspicion for a systemic disease causing EN, such as sarcoidosis. The diagnosis of DISR, in this case, was confirmed by biopsy‐proven noncaseating granulomas, which are consistent with sarcoidosis but require exclusion of other causes of granulomatous inflammation.

In most cases, DISRs are self‐limited and resolve upon discontinuation of the offending agent, as seen in this patient. The improvement of symptoms following the tapering of prednisone and the subsequent management of her condition suggest that stopping Dupixent was a crucial step in resolving the sarcoidosis‐like reaction. This aligns with existing literature where withdrawal of the causative drug often leads to symptom resolution without the need for further aggressive intervention [6]. Clinically, the improvement during drug withdrawal alongside the temporary connection between drug exposure and reactions, with no other causes, puts dupilumab as the probable potential cause for DISR [16].

To further assess the likelihood of dupilumab being the causative agent, we applied the NARANJO Adverse Drug Reaction Probability Scale. The patient scored in the “probable” category, suggesting that Dupilumab was likely responsible for the sarcoidosis‐like reaction. This standardized scoring system aids in objectively determining the causative relationship between a drug and an adverse event, reinforcing our hypothesis.

This case contributes to the growing body of evidence that suggests the need for vigilance in monitoring sarcoidosis‐like reactions in patients receiving immune‐modulating therapies, including newer agents like Dupixent. Clinicians should be aware of this potential adverse effect and consider DISR in the differential diagnosis when patients present unexplained granulomatous inflammation while on such medications. Further studies are needed to elucidate the mechanisms by which Dupixent and similar drugs may induce sarcoidosis‐like reactions and establish guidelines for managing these conditions effectively.

## Funding

No funding was received for this manuscript.

## Consent

No written consent has been obtained from the patients as there is no patient identifiable data included in this case report/series.

## Conflicts of Interest

The authors declare no conflicts of interest.

## Data Availability

The data that support the findings of this study are available on request from the corresponding author. The data are not publicly available due to privacy or ethical restrictions.
